# Validation of Two Questionnaires Assessing Nurses’ Perspectives on Addressing Psychological, Social, and Spiritual Challenges in Palliative Care Patients

**DOI:** 10.3390/nursrep14030179

**Published:** 2024-09-18

**Authors:** Vesna Antičević, Ana Ćurković, Linda Lušić Kalcina

**Affiliations:** 1 The University Department of Health Studies, University of Split, 21000 Split, Croatia; vanticev@ozs.unist.hr; 2 School of Medicine, University of Split, 21000 Split, Croatia

**Keywords:** palliative care, palliative medicine, psychosocial functioning, social support, spirituality, factor analysis

## Abstract

Background: Palliative care provides holistic support, addressing physical, psychological, social, emotional, and spiritual dimensions of suffering, known as “total pain”, to improve patients’ quality of life. Patients often rely on healthcare professionals, particularly nurses, for support. This study aimed to develop and validate questionnaires assessing nurses’ perceptions of psychological, social, and spiritual issues in palliative care and their effectiveness in managing them. Methods: Two self-rated questionnaires were created: the Psychological, Social, and Spiritual Problems of Palliative Patients’ Questionnaire and the Effectiveness in Coping with the Psychological, Social, and Spiritual Challenges of Palliative Care Patients. The study surveyed 237 nurses caring for palliative patients in Split Dalmatian County, Croatia. Results: The questionnaires demonstrated high reliability with Cronbach’s α values of 0.98 and 0.99. Factor analysis revealed four factors for the first questionnaire and three for the second. Nurses primarily perceived patients as experiencing fear and emotional/spiritual suffering, with the greatest difficulty coping with the fear of the disease outcomes. Nurses acknowledged the need for improvement in addressing patient challenges, highlighting gaps in the Croatian system. Conclusions: Ongoing efforts are crucial to prioritize palliative care globally, with nursing professionals playing a vital role in symptom management.

## 1. Introduction

The concept of palliative care is a comprehensive approach that considers an individual’s body, mind, and spirit. Palliative care focuses on addressing the “total pain”, which includes the physical, psychological, social, emotional, and spiritual dimensions of suffering [1]. The primary objective of palliative care is to enhance patients’ quality of life by meeting their complex and multidimensional needs, including their psychological, social, and spiritual requirements [2,3,4,5,6].

A systematic review conducted by Lormans et al. [5] and a meta-study by Edwards et al. [7] identified several social, psychological, and spiritual needs of palliative patients. According to their findings, palliative patients want to be treated as individuals and wish to retain control over their daily activities and decisions about their lives [5,7]. They require social support, communication, family support, and maintenance of relationships [5], as well as support, love, and security from their loved ones [7]. Palliative patients also have spiritual needs, such as the need to find meaning in life in the context of their illness and the need for a sense of belonging [5]. The results of the systematic review suggest the need to incorporate spiritual care into serious illness treatment, train interdisciplinary teams in spiritual care, and involve spiritual care specialists. Additionally, patient-centered approaches that recognize the health benefits of spiritual communities should be implemented, with increased awareness among health professionals and recognition of spirituality as a social health factor in research and community programs [8]. Palliative patients also require self-acceptance, acknowledgment, and peace. Faith in God has proved to be significant in easing their fears, drawing strength, and achieving peace [7]. Palliative patients seek support to cope with feelings of insecurity and imminent death [5], as well as dealing with anxiety, depression, grief, and anger [9]. Anxiety and depression are common responses to severe illness and the impending end of life [9,10]. Therefore, addressing the psychological needs of palliative patients aims to improve their overall psychological wellbeing and reduce distress [10], providing support to patients and their families [3]. Depression and psychological distress should not be considered normal occurrences in palliative patients [11], especially since they affect the experience of symptoms, pain, suffering, and mortality [11,12]. The literature suggests that spirituality in palliative care has favorable effects on patients [13]. Fulfilling spiritual needs can help patients accept their illness, cope with symptoms, and find purpose in life [14,15,16]. Studies have shown that meeting spiritual needs significantly affects the quality of life [17,18,19,20] and psychological state [17,21,22] of palliative patients, serving as a significant support in pain management [3]. In addition to psychological well-being and quality of life, severe illnesses can disrupt patient social functioning, limiting their autonomy and independence. Thus, addressing social needs provides patients with a sense of identity, belonging, and purpose, along with emotional and practical support [5].

Failure to meet these needs can lead to fear, insecurity, nervousness, panic attacks, anxiety [7], loss of hope, increased distress, and more frequent contemplation of hastening death among palliative patients [3]. Therefore, the importance of addressing these aspects of pain is emphasized in palliative care patients and in meeting their needs [5,11,17,23,24]. Patients often expect the help of healthcare professionals, especially nurses, to meet these needs. However, healthcare professionals often feel inadequately competent, which highlights the need for educating healthcare professionals, particularly nurses, on the needs of palliative patients and how to meet them [16,25].

When the needs of palliative patients are met, they can accept their illness and condition, thus coping with death more easily [26]. A larger number of studies confirm the importance of recognizing psychological, social, and spiritual issues in palliative care patients [3,25,27]. However, a review of the literature did not identify a validated measure for assessing these problems.

By reviewing the available literature and screening tools on palliative care, we found several different questionnaires (e.g., FICA [28], HOPE [29], SPIRIT [30], SNAP [31], NAT: PD-C [32]) that individually measure various aspects of the lives and needs of palliative patients. However, most of these questionnaires do not simultaneously address the psychological, social, and spiritual needs of palliative patients, as our study does. Additionally, most of these tools are self-assessment questionnaires where palliative patients evaluate their own difficulties, whereas in our study, the questionnaires are designed for healthcare workers (nurses) to assess the needs and satisfaction of these needs in palliative care patients.

The Croatian Chamber of Nurses has adopted competencies for nurses in palliative care from the European Association for Palliative Care. According to these competencies, a nurse provides specialized care, identifies and responds to the patient’s psychological, social, and spiritual needs, and engages in teamwork within an interdisciplinary team [33]. The interdisciplinary palliative care team includes a physician, nurses, a social worker, a psychologist, and a spiritual advisor [34].

Therefore, the purpose of this study was to create and validate two questionnaires (Supplementary: File S1: Questionnaires) measuring nurses’ perceptions of the psychological, social, and spiritual issues faced by palliative patients, as well as the nurses’ perceptions of the patients’ effectiveness in coping with these challenges. Additionally, this study aimed to investigate nurses’ perspectives regarding the level of psychological, social, and spiritual problems faced by palliative patients. Finally, the study aimed to understand how nurses perceive patients’ effectiveness in coping with disease-related challenges, including coping with the fear of the consequences of the disease, spiritual and daily challenges, and connection with loved ones.

## 2. Materials and Methods

### 2.1. Study Design

The study was conducted as a cross-sectional validation study. Two novel questionnaires were designed to assess the psychological, social, and spiritual challenges faced by palliative patients. The study targeted a convenient sample of nurses providing care to palliative patients. The questionnaires were administered online via Google Forms to ensure broad accessibility. The development and validation of these questionnaires were conducted in four distinct phases, as detailed below.

### 2.2. Questionnaire Development and Validation

The design and development of the questionnaires occurred in four phases. The total score for each questionnaire, which ranges from 0 to 265, reflects the extent of the psychological, social, and spiritual challenges of patients as perceived by nurses. The first three phases were conducted jointly, while the fourth phase focused on separate validation procedures for each.

#### 2.2.1. Item Development

**Figure 1:**
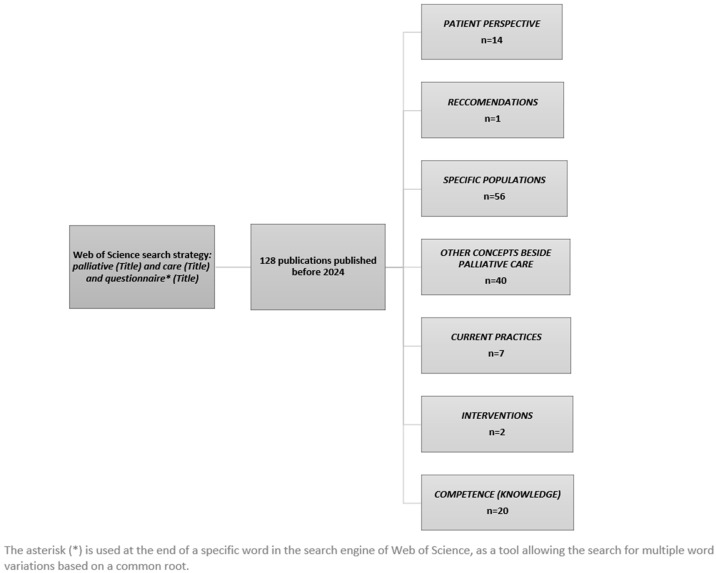
Flow chart showing procedure of selecting relevant studies.

In the first phase, we conducted a literature review by searching the Web of Science platform using the following search strategy: palliative (Title) and care (Title) and questionnaire * (Title). The strategy resulted in a total of 128 scientific publications on the relevant topics, without narrowing the publications to recent decades of publications. The procedures for accessing, screening, and selecting the studies were related to the type of article and the topic of relevance. All publications were separated into the following categories: patient perspective, recommendations, a specific population, other concepts besides palliative care, current practices, interventions, and competence (knowledge). We associated each topic in the publications with the aforementioned categories (Figure 1). Finally, out of the list, four relevant publications, Pruthi et al. [35], Sewtz et al. [36], A-Ansari et al. [37], Osse et al. [38], remained for the development of the questionnaire used in this study. The aforementioned publications thoroughly represent the subject investigated in our research, and these publications were not constrained to specific topics defined in the flow chart.

This review identified the most common psychological, social, and spiritual needs of palliative patients. In determining spiritual needs, we were also guided by the European Association for Palliative Care (EAPC) consensus-based definition: ‘Spirituality (or a sense of meaning and purpose) is the dynamic dimension of human life that relates to the way persons (individual and community) experience, express and/or seek meaning, purpose and transcendence, and the way they connect to the moment, to self, to others, to nature, to the significant and/or the sacred where spirituality is expressed through beliefs, values, traditions, and practices’ [39]. In the second phase, an expert team reviewed and adjusted the questionnaire items to ensure content validity, measurability, and linguistic suitability. Items deemed less understandable were revised or removed. In the third phase, a double translation method was employed as most items were in English. Each item was independently translated into Croatian by team members, resulting in a final list of 53 items. These were then back-translated by an English professor to ensure accuracy and meaning preservation. Any discrepancies were resolved through mutual agreement. In the fourth phase, the 53 items were divided into two questionnaires, and validation was conducted for each questionnaire separately.

#### 2.2.2. Content Validity

In the second phase of questionnaire development, an expert focus group was convened to review and adjust the questionnaire items to ensure the content validity, measurability, and linguistic suitability. The focus group consisted of professionals who are subject matter experts in their respective domains, including a nurse, a bioethics expert, a clinical psychologist, a priest, and a sociologist. The criteria for selecting the experts for focus group members were described in the Section 1. The process involved multiple rounds of structured group discussions. The members of the focus group provided feedback on the items, which were revised based on their expert judgment to achieve a final list of 53 items. Any discrepancies in the interpretation of items were resolved through mutual agreement, ensuring a reliable and well-validated set of questionnaire items.

According to the Biopsychosocial–Spiritual model [40], holistic health care must address the patient’s physical, psychological, social, and spiritual wellbeing [41], and this requires the collaboration of a multidisciplinary palliative care team, such as the one included in this research. Psychologists are essential for addressing the emotional and mental wellbeing of patients, helping them cope with anxiety, depression, and fear related to their condition. Nurses provide essential physical care and daily support, ensuring the patient’s comfort and attending to medical needs. Sociologists contribute by considering the patient’s social environment, family dynamics, and community support, all of which play a crucial role in their wellbeing. Priests or spiritual advisors offer guidance for patients seeking meaning, peace, or closure during their final stages of life, addressing their spiritual needs. As ethical issues in palliative care are becoming more frequent, especially regarding patient rights, dignity, and the appropriateness of certain treatments, it is important to have a bioethics expert on the team. Such an expert helps in navigating complex ethical challenges in palliative care, ensuring that care respects the patient’s values, beliefs, and legal rights, while also guiding healthcare professionals in making informed and ethically sound decisions [42]. Experts included in the focus group ensured the inclusion of comprehensive ethically guided items investigating patients’ needs at the end of life.

#### 2.2.3. Factor Analysis

Although both questionnaires consisted of content-identical items, factor analysis showed that they were different questionnaires that measure similar but not identical aspects of the patient’s life. Therefore, the validation process for each questionnaire was described separately.

### 2.3. Participants Selection and Data Collection

The research was carried out on a convenient sample of 237 female nurses who provide care to palliative patients. The inclusion criteria were being a nurse and having experience in palliative care. We defined experience in palliative care according to the recommendations of Janice Monti [43], suggesting that palliative care nurses provide curative care for critically ill patients and comfort for the terminally ill, while supporting families with advice and bereavement care. They work with patients of all ages, treating conditions like cancer, kidney failure, heart disease, and dementia, as well as life-threatening injuries or disabilities requiring ongoing care. All the nurses were confirmed to have provided such care at any level of their personal work experience, and it was not mandatory that they be currently working solely in palliative care.

Therefore, all respondents in the current study had the aforementioned experience, and they had a nursing education of level 4.2 or higher, according to the Croatian Qualifications Framework. This is a mandatory level of education for qualified nurses in Croatia. On the other hand, the experience in palliative care was not defined in years of palliative care experience but was solely a self-reported measure in terms of affirmative answer to the question: Do you have experience in working with palliative care patients? Additionally, we also asked our respondents about the overall length of their experience (in years).

Among them, 65.8% cared for palliative patients as part of their daily work tasks. Most of them were employed at the University Hospital of Split (about 85%), while the others were employees of The Split Health Centre, including hospice and nursing homes. On average, they had 10.7 years of work experience with palliative patients. Both questionnaires were constructed as self-rated questionnaires consisting of 53 items, with 5-point Likert scale responses. Self-administered questionnaires were applied online via Google form during September 2023. Participants were asked to assess the prevalence of patients’ problems and their adequacy in addressing these in palliative care settings. At the start of the questionnaire, there were initial questions regarding the experience with palliative patients, education in this field, and a self-assessment of competence in working with palliative patients.

### 2.4. Statistical Analysis

Descriptive data on socio-demographic sample’ characteristics were reported as the mean and standard deviation or the frequency and percentage, depending on the variables being reported. Since all the variables did not pass the Shapiro–Wilk normality test, we also used the Kolmogorov–Smirnov test, which is less powerful. Since all the variables were not distributed normally, we decided to use parametric analysis following the assessment of the Q-Q plots and the stem-and-leaf plots. This was based on the reports indicating that the adequate control for Type I error might be achieved with a total sample size of at least 200, even in skewed populations [44].

Exploratory factor analysis was conducted to determine the questionnaire’s factorial structure and explore which items collectively constituted a particular construct [45]. The Kaiser–Meyer–Olkin and Bartlett’s test of sphericity were both used to assess the suitability of data for factor analysis. Since the questionnaires used an ordinal measure of a five-point Likert scale response, we calculated the correlation matrix among items and established the Eigenvalues to determine the optimum number of factors to be extracted. We used a parallel analysis scree plot to visualize the number of factors we needed to obtain. The exploratory factor analyses were achieved by using the maximum likelihood factor extraction method with the Varimax rotation with Kaiser normalization. We extracted the factors based on not only factor loading but also the interpretability of the factors. Descriptive indicators (mean and standard deviation) were used as measures of the average responses to each item individually and for each factor on both questionnaires.

### 2.5. Ethical Considerations

This research was conducted under the institutional project “Assessment of Psychological and Spiritual Issues in Palliative Patients in the Split-Dalmatia County” (SOZS-IP-2022-1). The research was approved by the Ethics Committees of the University Hospital of Split (Class: 500-03/22-01/167 No: 2181-147/01/06/M.5.-22-02. September 2022), the Split Health Centre (Class: 053-01/22-01l/021 No: 2181-149-18-22-00. September 2022), and the University Department of Health Studies (Class: 029-03/22-08/01 No: 2181-228-103/1-22-32. October 2022). Participation was voluntary and anonymous with the possibility of refusing to participate or withdrawing at any time after consenting. The participants were informed about who was conducting the research, as well as the purpose and objectives of the study. Since research the was conducted online, pressing the “Submit” button was considered consent to participate in the research. Instructions for completing the questionnaires were provided to ensure the accuracy of the data.

## 3. Results

### 3.1. Socio-Demographic Sample Characteristics

The current study included 237 nurses, all female, with an average experience of 10.7 ± 10.8 years. Among them, 162 (68.4%) nurses were educated in palliative care during their formal nursing education, and 92 (38.8%) were continuously being educated in palliative care via lifelong learning educational processes. In Croatia, there is no specialization for nurses in the field of palliative care. The nurses in our sample were of a general profile, and all had experience working with palliative patients as part of their job positions. The nurses answered the questions having in mind palliative patients of different diagnoses who had preserved cognitive abilities.

### 3.2. Psychological, Social, and Spiritual Needs of Palliative Patients Questionnaire

#### 3.2.1. Content and Face Validity

The Kaiser–Meyer–Olkin measure of sampling adequacy was 0.952, and Bartlett’s test of sphericity reached statistical significance (*p* < 0.001), which suggested that these data are very suitable for factor analysis [46,47] (Table 1). Based on the percentages of explained variance (Table 2) and the value of the scree plot, complemented by the context of this study, it was decided to retain four factors including 53 items, explaining 64.56% of the total item variance in the database. Factor I (22 items), Emotional and spiritual suffering, accounted for 49.65% of the variance; Factor II (13 items), Fear of the consequences of the disease, accounted for 6.852%; Factor III (9 items), Social support, accounted for 4.76%; Factor IV (9 items), General functionality, accounted for 3.31%. Factor I explains the highest percentage of variance indicating that Emotional and spiritual suffering is the most influential factor (Table 2).

#### 3.2.2. Construct Validity and Reliability

The reliability coefficient indicates the high reliability of the questionnaire with a value of Cronbach α = 0.98.

### 3.3. Effectiveness in Coping with the Psychological, Social, and Spiritual Challenges of Palliative Care Patients

#### 3.3.1. Content and Face Validity

The high Kaiser–Meyer–Olkin value (0.963) and the significant result from Bartlett’s Test (*p*-value < 0.001) both suggest that the present data are highly suitable for factor analysis. The variables in this dataset are sufficiently correlated, supporting the assumption that there are underlying factors that can be extracted (Table 3).

This factor analysis indicates a three-factor solution. Factor I, Coping with the fear of the consequences of the disease, appears to be the most dominant, explaining a substantial amount of variance (62.62%). Factors II, Spirituality and the purpose of life, and III, Connection with loved ones, contribute less to the overall variance (3.85% and 3.5%, respectively) but still play an important role in explaining nurses’ perceptions. The cumulative percentages suggest that the three factors explain almost 70% of the total variance in the data. The use of Varimax rotation with Kaiser normalization suggests that the factors are relatively uncorrelated, simplifying the interpretation of the underlying structure (Table 4).

#### 3.3.2. Construct Validity and Reliability

A Cronbach’s Alpha of 0.985 is indicative of a very reliable set of items. It suggests a high degree of internal consistency, meaning that the items in the questionnaire are measuring the same underlying construct consistently.

### 3.4. Nurses Assessment of the Psychological, Social, and Spiritual Needs of Palliative Patients’ Questionnaire and Effectiveness in Coping with the Psychological, Social, and Spiritual Challenges of Palliative Care Patients

**Figure 2:**
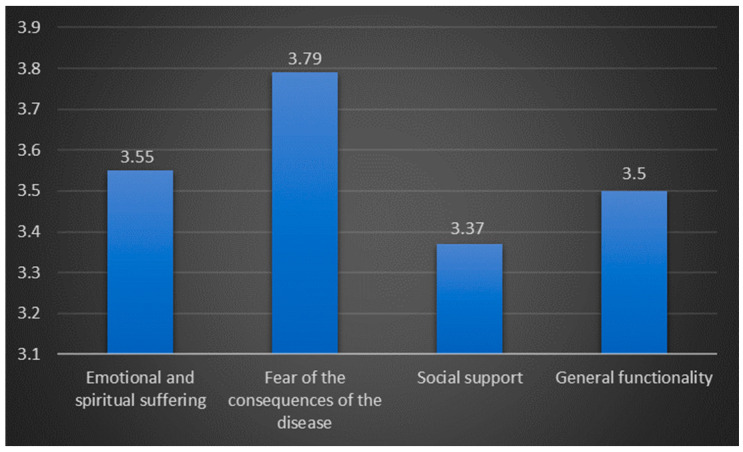
Psychological, social, and spiritual problems of palliative patients.

**Figure 3:**
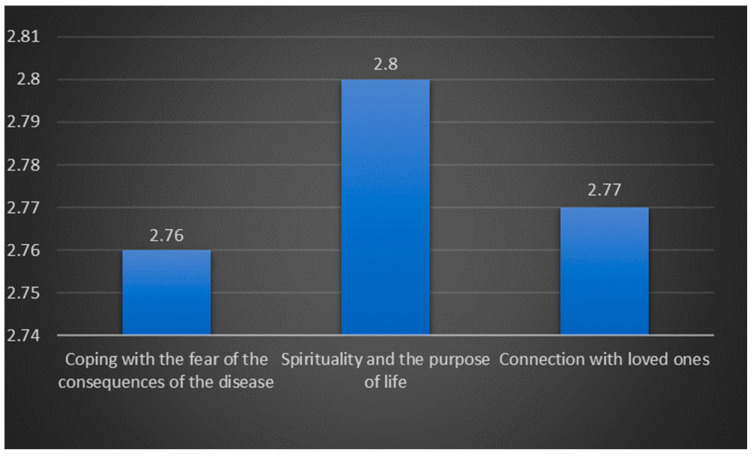
Effectiveness of coping with psychological, social, and spiritual problems of palliative patients.

To assess nurses’ perceptions of the psychological, social, and spiritual needs and effectiveness in coping with the disease-related challenges of palliative care patients, we calculated the mean scores and standard deviations for each item separately and a total mean score for each factor (Figure 2 and Figure 3). The results reveal that nurses perceive palliative care patients to predominantly experience fear related to their illness’s outcomes, as well as emotional and spiritual suffering. Accordingly, they have the hardest time facing the fear of disease consequences. Although a direct comparison between the two utilized questionnaires is not feasible, the data suggest that nurses believe the recognized problems of patients are inadequately addressed. On average, they note that patients experience various psychological, social, and spiritual problems occasionally to frequently, with scores ranging from 3.37 to 3.79. However, the effectiveness of dealing with problems related to the disease is generally rated as rarely to occasionally, with average scores ranging from 2.76 to 2.80, indicating a significant gap in meeting these crucial needs.

## 4. Discussion

This study introduces two validated questionnaires, offering valuable insights into nurses’ perceptions of patients’ fear and emotional/spiritual suffering, particularly their struggles with the fear of the disease outcomes. As most previous research has been qualitative, these questionnaires serve as a beneficial addition to the field. The exploratory factor analysis results shed light on key factors influencing nurses’ perceptions about the problems and the effectiveness of the coping strategies at the end of life. This points to the need to develop targeted interventions and training programs for palliative care teams, aiming to improve their understanding and management of patients’ psychological, social, and spiritual suffering. The identified gap between the perceived problems of patients and the effectiveness in addressing them highlights the need for enhanced strategies and resources. This includes increased interdisciplinary collaboration, ongoing post-graduate education, and focused attention on addressing fears, providing spiritual support, promoting social connections, and ensuring overall functionality in palliative care.

Four key factors of the first questionnaire, Emotional and spiritual suffering, Fear of disease consequences, Social support, and General functionality, together explain about 65% of the total variance. These extracted factors confirm that nurses in this study assess the problems of their patients in accordance with Saunders’s [48] views of the concept of ‘total pain’, often experienced by palliative patients, which includes physical, social, emotional, and spiritual distress.

A three-factor solution identified for the second questionnaire of Coping with fear of disease consequences, Spirituality and the purpose of life, and Connection with loved ones explains almost 70% of the total variance in the data reinforcing its importance in capturing the complexity of nurses’ perceptions regarding patients’ coping with disease-related challenges.

Based on nurses’ perceptions, the most challenging issues faced by palliative care patients are fear of the outcomes of their illness, emotional distress, and spiritual struggles, according to the total mean values. More specifically, nurses highlight that palliative care patients have the most difficulty facing the fear of the disease consequences. This suggests that addressing and alleviating the psychological impact of the disease, particularly the fear associated with potential outcomes, is a significant challenge in palliative care. This finding confirmed the previous research explaining that the suffering of palliative patients exists on a continuum resulting from various sources: grief about current and anticipated losses, fear and uncertainty about the future, unresolved issues from the past, spiritual issues, and concerns about loved ones [49]. Further, in line with previous research, it is also known that it is common in palliative care to experience spiritual distress, defined as the disruption of one’s beliefs or value system suggesting that spiritual distress happens when a person is unable to find sources of meaning, love, and comfort or when conflict occurs between beliefs and life’s events [50].

Nurses recognized issues in palliative care as inadequately addressed, indicating a potential gap in care delivery for patients’ psychological and spiritual problems. It is particularly worrisome that nurses rate patients’ effectiveness in dealing with disease-related problems with lower scores (rarely to occasionally), especially about difficulties related to the fear of the consequences of the disease.

The results of this study indicate that nurses working with palliative patients recognize that psychological, social, and spiritual distress are common in end-of-life patients. This often triggers thoughts of hastening death; hence, patients can become demoralized and hopeless, as suggested by Moss and Dobson [51]. Nurses in this study are also aware that there is room for improvement in the Croatian palliative care system. This issue also seems to arise on a global scale. Although palliative care primarily aims to minimize physical suffering and thus improve the quality of the patient’s life [52], the psychosocial wellbeing of the patient must also be addressed. Patients at the end of their lives are often faced with physical and emotional pain and need to preserve their dignity and a sense of control, where healthcare professionals need to recognize and respect these needs and provide an appropriate response. According to Sherman et al. [53], even though there are positive developments in the global landscape of palliative care, challenges such as disparities in access, resource and education limitations, and varying cultural attitudes toward end-of-life care persist. Inadequate training, insufficient compensation, and personal discomfort with death among healthcare workers are mentioned as the main deficiencies in the delivery of quality palliative care [54].

The results of this study carry significant practical implications. They suggest that enhancing the quality of palliative care necessitates the implementation of targeted interventions, comprehensive training programs, and fostering increased collaboration. Additionally, future research should consider the perspectives of patients, families, and other healthcare professionals to gain a holistic understanding of coping mechanisms and the effectiveness of palliative care. By prioritizing palliative care globally and investing in the professional development of nursing professionals, we can strive to offer compassionate and comprehensive care to individuals facing life-limiting illnesses.

This study also has several limitations related to the methodological approach. First, the study used a convenient sample of nurses from a single geographic area (mainly the University Hospital of Split), which may limit the generalizability of the findings to other regions or healthcare settings. All the participants were female, indicating a potential gender bias in the validation process. This may affect the applicability of the questionnaires to male nurses’ perspectives. Second, while we conducted exploratory factor analysis, we did not perform confirmatory factor analysis, which could have further validated the factor structure of the questionnaires. In addition, we did not establish convergent validity by comparing our questionnaires with existing validated measures of similar constructs. Next, the cross-sectional nature of the study limits our ability to assess how nurses’ perceptions might change over time or with increased experience in palliative care. Finally, the questionnaires were validated only from the nurses’ perspective, without input from patients, families, or other healthcare professionals involved in palliative care. Generally, using self-assessment instruments has limitations such as capturing only the current state, which may not reflect a permanent condition, the inability to control the sincerity of responses, the provision of socially desirable answers, etc. Except for the limitations mentioned, there are also some specific challenges and constraints when healthcare professionals (like nurses) use this kind of instrument to assess patients’ needs. These could include biases, resulting from limitations in nurses’ understanding of the patient’s personal experiences, difficulty in capturing the full depth of a patient’s subjective experience, and limitations in the communication between patient and nurse, especially in sensitive areas such as spirituality and psychological distress. These limitations should be taken into consideration when planning similar studies. A possible solution for future implementation could be training programs for healthcare providers to better interpret and apply these tools. Therefore, our study not only aims to address the gap by creating instruments designed for healthcare professionals but also acknowledges the need for ongoing training and further research aimed at developing complementary methods that can bridge these gaps in understanding and application.

## 5. Conclusions

In this study, we successfully developed and validated two novel questionnaires for assessing psychological, social, and spiritual aspects of palliative care from nurses’ perspectives. The validation process demonstrated strong psychometric properties. Exploratory factor analysis revealed a four-factor structure for the first questionnaire and a three-factor structure for the second, explaining 64.56% and 70% of the total variance, respectively. This suggests that both questionnaires capture distinct and meaningful dimensions of palliative care. The development process, including a literature review, expert focus groups, and a double translation method, ensured the content validity for both questionnaires. Both questionnaires demonstrated excellent internal consistency, with Cronbach’s alpha values of 0.98 and 0.985, indicating high reliability of the items within each scale.

To conclude, there is an urgent need to enhance the care provided to palliative patients in the Croatian healthcare system. Nurses, who are at the forefront of caregiving, play a crucial role in managing patients’ symptoms and addressing their psychological, social, and spiritual needs. However, the study reveals that there is a significant gap in meeting these needs, especially in addressing patients’ emotional and spiritual struggles and fears.

## Figures and Tables

**Table 1:** Kaiser–Meyer–Olkin measure and Bartlett’s Test of Sphericity for factor analysis suitability.

Kaiser–Meyer–Olkin Measure of Sampling Adequacy		0.952
Bartlett’s Test of Sphericity	Approx. Chi-Square	11.455.736
	df	1.378
	*p*	<0.001

**Table 2:** Factor analysis of Psychological, Social, and Spiritual Needs of Palliative Patients questionnaire.

In Your Opinion, to What Extent Do Most Palliative Patients Experience These Problems?	I Emotional and Spiritual Suffering	II Fear of the Consequences of the Disease	III Social Support	IV General Functionality
Initial Eigenvalues	26.31	3.63	2.52	1.75
% of Variance	49.65%	6.85%	4.76%	3.31%
Cumulative %	49.65%	56.5%	61.26%	64.56%
Rotation Sums of Squared	10.7	3	9.41	5.01
% of Variance	20.19%	17.76%	9.46%	8.13%
Cumulative %	20.19%	37.96%	47.42%	55.55%
Item Loadings				
P1				0.497
P2				0.585
P3				0.625
P4				0.735
P5				0.771
P6				0.605
P7				0.728
P8				0.677
P9				0.515
P10			0.491	
P11			0.516	
P12			0.450	
P13			0.617	
P14			0.692	
P15			0.594	
P16			0.616	
P17			0.613	
P18		0.741		
P19		0.518		
P20		0.709		
P21		0.766		
P22		0.800		
P23		0.609		
P24		0.827		
P25		0.781		
P26		0.779		
P27		0.702		
P28	0.502			
P29	0.446			
P30	0.492			
P31	0.516			
P32		0.552		
P33	0.562			
P34	0.570			
P35	0.578			
P36	0.579			
P37			0.432	
P38	0.562			
P39		0.574		
P40	0.606			
P41	0.620			
P42	0.611			
P43	0.710			
P44	0.728			
P45	0.694			
P46	0.778			
P47	0.720			
P48	0.740			
P49	0.663			
P50		0.565		
P51	0.632			
P52	0.721			
P53	0.713			

**Table 3:** Kaiser–Meyer–Olkin measure of sampling adequacy and Bartlett’s Test of Sphericity.

Kaiser–Meyer–Olkin Measure of Sampling Adequacy		0.963
Bartlett’s Test of Sphericity	Approx. Chi-Square	14.189.599
	df	1.378
	*p*	<0.001

**Table 4:** Factor analysis of effectiveness in coping with the psychological, social, and spiritual challenges of palliative care patients.

In Your Opinion, to What Extent Are the Psychological, Social, and Spiritual Problems of Palliative Patients Being Addressed?	I Coping with the Fear of the Consequences of the Disease	II Spirituality and the Purpose of Life	III Connection with Loved Ones
Initial Eigenvalues	33.190	2.039	1.853
% of Variance	62.623	3.848	3.496
Cumulative %	62.623	66.470	69.966
Rotation Sums of Squared	12.934	12.084	12.064
% of Variance	24.404	22.801	22.762
Cumulative %	24.404	47.204	69.966
Item Loadings			
SN1	0.815		
SN2	0.808		
SN3	0.789		
SN4	0.781		
SN5.	0.723		
SN6	0.674		
SN7	0.673		
SN8	0.662		
SN9	0.657		
SN10	0.634		
SN11	0.630		
SN12	0.629		
SN13	0.608		
SN14	0.583		
SN15	0.581		
SN16	0.570		
SN17		0.531	
SN18		0.777	
SN19		0.719	
SN20		0.691	
SN21		0.676	
SN22		0.666	
SN23		0.665	
SN24		0.655	
SN25		0.652	
SN26		0.650	
SN27		0.648	
SN28		0.637	
SN29		0.627	
SN30		0.623	
SN31	0.547		
SN32		0.567	
SN33		0.563	
SN34		0.553	
SN35		0.511	
SN36			0.709
SN37			0.692
SN38			0.690
SN39			0.688
SN40			0.684
SN41			0.680
SN42			0.657
SN43			0.650
SN44			0.640
SN45			0.629
SN46			0.624
SN47			0.616
SN48			0.612
SN49			0.610
SN50			0.577
SN51			0.545
SN52			0.354
SN53			0.344

## Data Availability

The data that support the findings of this study are available from the corresponding author upon reasonable request.

## Supplementary Materials

The following supporting information can be downloaded at: https://www.mdpi.com/article/10.3390/nursrep14030179/s1. File S1: Questionnaires.

## Public Involvement Statement

There was no public involvement in any aspect of this research.

## Guidelines and Standards Statement

This manuscript was drafted against the STROBE Statement—Checklist of items that should be included in reports of cross-sectional studies.
