# Supplementary material for: Validation of Two Questionnaires Assessing Nurses’ Perspectives on Addressing Psychological, Social, and Spiritual Challenges in Palliative Care Patients
Source: Nurs Rep. 2024 Sep 18;14(3):2415–29. doi: 10.3390/nursrep14030179 (PMC11417697; doi:10.3390/nursrep14030179)
Supplement: Supplementary file 1 [file nursrep-14-00179-s001.zip › nursrep-3093672-supplementary.pdf]

Upitnik psiholoških, duhovnih i socijalnih potreba palijativnih bolesnika

Upitnik učinkovitosti u nošenju s psihološkim, duhovnim i socijalnim potrebama palijativnih bolesnika

Cilj ovog istraživanja je ispitati percepciju medicinskih sestara o potrebama i zadovoljenosti potreba palijativnih pacijenata. Istraživanje je potpuno anonimno te je Vaše sudjelovanje dobrovoljno i možete odustati u bilo kojem trenutku. Popunjavanjem upitnika smatra se da pristajete na sudjelovanje u istraživanju.

U upitnicima koriste se izrazi koji imaju rodno značenje, neovisno o tome koriste li se u muškom ili ženskom rodu, neutralni su i obuhvaćaju na jednak način muški i ženski rod.

Upute za popunjavanje upitnika:

Ovi upitnici namijenjeni su medicinskim sestrama koje rade s palijativnim bolesnicima. Molimo Vas da se Vaši odgovori odnose samo na rad s bolesnicima koji boluju od:

- akutnih, ozbiljnih i po život opasnih bolesti kod kojih je liječenje vrlo dugotrajno, zahtjevno i popraćeno znatnim teškoćama, a izlječenje ipak moguće;
- progresivnih kroničnih bolesti i stanja (maligne bolesti, kronične bolesti), demencije i drugih bolesti koje uzrokuju potpunu nesamostalnost u zadovoljavanju osnovnih ljudskih potreba;
- stanja uzrokovanih teškim ozljedama i traumama;
- bolesti čiji je stadij u posljednjoj fazi (demencija, završni stadij karcinoma, druge bolesti ili teški invaliditet) kod kojih oporavak nije moguć pa je skrb neizostavna do kraja života.

Vaše zanimanje ___________________________

Institucija/odjel u kojem ste zaposleni _________________________________

Iskustvo rada s palijativnim bolesnicima (broj godina) ________________

U prvom dijelu se pitanja odnose na vaš doživljaj i iskustvo rada s palijativnim bolesnicima. Molimo vas da na pitanja odgovorite sa DA ili NE.

1. Rad s palijativnim bolesnicima dio je mojih redovitih radnih zadataka. DA NE
2. Tijekom mog formalnog obrazovanja slušala sam nastavu i polagala ispite iz područja palijativne skrbi. DA NE
3. Nakon završenog formalnog obrazovanja sudjelovala sam u programima cjeloživotnog učenja iz područja palijativne skrbi DA NE
4. Smatram se dovoljno educiranom iz područja palijative. DA NE
5. Rad s palijativnim bolesnicima za mene je vrlo emocionalno iscrpljujući te mi i samoj ponekad treba pomoć kako bih bolje odgovorila na probleme/potrebe palijativnih bolesnika. DA NE
6. Stručnjacima koji se bave palijativnom skrbi, zbog emocionalne zahtjevnosti posla, potrebno je osigurati različite oblike podrške, uključujući psihološku pomoć DA NE
7. Smatram da je u programima srednjih zdravstvenih škola i fakulteta potrebno uvesti više predmeta iz područja palijativne medicine. DA NE
8. Smatram da je češće potrebno organizirati skupove na kojima će se prezentirati znanja iz područja palijativne medicine. DA NE

9. Palijativnom skrbi se treba baviti interdisciplinarni tim stručnjaka (npr. liječnici, medicinske sestre, psiholozi, svećenici, socijalni radnici i sl.) DA NE
10. U našem sustavu zdravstvene i socijalne skrbi postoje timovi za palijativnu skrb koji su dostatni za skrb o palijativnim bolesnicima DA NE
11. Briga o potrebama palijativnih bolesnika najviše je prepuštena obiteljima DA NE

Drugi dio: Molim Vas da u drugom stupcu odgovorite u kojoj mjeri većina osoba kojoj se pruža palijativna skrb doživljava poteškoće odnosno ima potrebe u različitim područjima svog života. Zaokružite broj (od 1 do 5) koji odražava **Vaše mišljenje** o izraženosti svake navedene teškoće.

U drugom stupcu zaokružite broj (od 1 do 5) koji odražava **Vaše mišljenje** o tome u kojoj mjeri su navedene potrebe zadovoljene?

**Poteškoće/potrebe**	U kojoj mjeri većina palijativnih bolesnika doživljava poteškoće odnosno ima potrebe u različitim područjima svog života?<br>1- uopće nije problem<br>2- poteškoća/potreba je prisutna u manjoj mjeri<br>3- poteškoća/potreba je umjereno izražena<br>4- poteškoća/potreba je vrlo izražena<br>5- to je najveća poteškoća/nezadovoljena potreba većine korisnika palijativne skrbi	U kojoj mjeri su potrebe kod većine palijativnih bolesnika zadovoljene?<br>1-uopće nije zadovoljena<br>2- u manjoj mjeri je zadovoljena<br>3- potreba je zadovoljena u umjerenom stupnju<br>4- u većoj mjeri je zadovoljena<br>5- u potpunosti je zadovoljena
1. Poteškoće u osmišljavanju dana.	1 2 3 4 5	1 2 3 4 5
2. Poteškoće u opuštanju/relaksaciji.	1 2 3 4 5	1 2 3 4 5
3. Poteškoće pri zapošljavanju ili nastavku studiranja (ne odnosi se na bolesnike s demencijom ili u terminalnom stadiju).	1 2 3 4 5	1 2 3 4 5
4. Poteškoće u brizi za djecu ili čuvanju djece. (ne odnosi se na bolesnike s demencijom ili u terminalnom stadiju).	1 2 3 4 5	1 2 3 4 5
5.Problemi u povezanosti s partnerom.	1 2 3 4 5	1 2 3 4 5
6.. Poteškoće u razgovoru o bolesti s partnerom..	1 2 3 4 5	1 2 3 4 5
7. Problemi u odnosu s djecom (ili samo jednim djetetom.	1 2 3 4 5	1 2 3 4 5
8. Poteškoće u odnosu s obitelji, prijateljima, susjedima ili kolegama.	1 2 3 4 5	1 2 3 4 5
9. Teško mu (joj) je razgovarati o bolesti jer ne želi opteretiti druge.	1 2 3 4 5	1 2 3 4 5

10. Smatra da drugi nisu spremni razgovarati o bolesti.	1 2 3 4 5	1 2 3 4 5
11. Različita mišljenja glede vrste liječenja koje treba biti primijenjeno.	1 2 3 4 5	1 2 3 4 5
12. Doživljava premalo podrške od drugih.	1 2 3 4 5	1 2 3 4 5
13. Teškoće u pronalaženju povjerljive osobe s kojom može razgovarati o bolesti.	1 2 3 4 5	1 2 3 4 5
14. Dobiva premalo praktične pomoći od partnera ili obitelji.	1 2 3 4 5	1 2 3 4 5
15. Drugi ljudi se ponašaju prezabrinuto.	1 2 3 4 5	1 2 3 4 5
16. Drugi značajni ljudi dramatiziraju situaciju.	1 2 3 4 5	1 2 3 4 5
17. Drugi ljudi poriču ozbiljnost situacije.	1 2 3 4 5	1 2 3 4 5
18.Usamljenost.	1 2 3 4 5	1 2 3 4 5
19. Drugi ljudi su ga napustili.	1 2 3 4 5	1 2 3 4 5
20.Depresivno raspoloženje.	1 2 3 4 5	1 2 3 4 5
21. Više ne osjeća zadovoljstvo u ničemu.	1 2 3 4 5	1 2 3 4 5
22. Strah od tjelesne patnje.	1 2 3 4 5	1 2 3 4 5
23. Strah od medicinskog tretmana.	1 2 3 4 5	1 2 3 4 5
24. Strah od pogoršanja bolesti.	1 2 3 4 5	1 2 3 4 5
25. Strah od samoće.	1 2 3 4 5	1 2 3 4 5
26. Strah od smrti.	1 2 3 4 5	1 2 3 4 5
27. Poteškoće pri nošenju s nepredvidljivošću budućnosti.	1 2 3 4 5	1 2 3 4 5
28. Poteškoće pri iskazivanju emocija.	1 2 3 4 5	1 2 3 4 5
29. Osjećaj krivnje.	1 2 3 4 5	1 2 3 4 5
30.. Osjećaj srama.	1 2 3 4 5	1 2 3 4 5
31. Gubitak kontrole nad emocijama.	1 2 3 4 5	1 2 3 4 5
32.. Poteškoće u prihvaćanju promjena tjelesnog izgleda.	1 2 3 4 5	1 2 3 4 5
33.Poteškoće u sagledavanju pozitivnih strana situacije.	1 2 3 4 5	1 2 3 4 5
34. Osjećaj preopterećenosti zbog svih odluka koje se moraju donijeti.	1 2 3 4 5	1 2 3 4 5

35. Poteškoće pri angažiranju u korisne svrhe..	1 2 3 4 5	1 2 3 4 5
36.. Teško mu je biti dostupan drugima.	1 2 3 4 5	1 2 3 4 5
37. Poteškoće u zadržavanju povjerenja u Boga ili u religiju.	1 2 3 4 5	1 2 3 4 5
38. Poteškoće u pronalaženju smisla smrti.	1 2 3 4 5	1 2 3 4 5
39. Poteškoće pri prihvaćanju bolesti.	1 2 3 4 5	1 2 3 4 5
40 Poteškoće u prihvaćanju izgubljenog vremena ili prilika u životu.	1 2 3 4 5	1 2 3 4 5
41. Poteškoće u prihvaćanju krivih životnih poteza koji se ne mogu ispraviti.	1 2 3 4 5	1 2 3 4 5
42. Poteškoće zbog nemogućnosti pomirenja s nekim ljudima koji su prošli kroz njihov život.	1 2 3 4 5	1 2 3 4 5
43. Poteškoće u traženju duhovne asistencije zbog straha od smrti.	1 2 3 4 5	1 2 3 4 5
44. Poteškoće u primanju sakramenta bolesničkog pomazanja zbog povezivanja istog s posljednjom pomašću tj. skorom smrću.	1 2 3 4 5	1 2 3 4 5
45. Poteškoće u obavljanju uobičajenih aktivnosti.	1 2 3 4 5	1 2 3 4 5
46. Poteškoće pri nastavku obavljanja društvenih aktivnosti.	1 2 3 4 5	1 2 3 4 5
47. Poteškoće u prepuštanju poslova drugima zbog nemogućnosti daljnjeg obavljanja istih.	1 2 3 4 5	1 2 3 4 5
48. Poteškoće zbog ovisnosti o drugima.	1 2 3 4 5	1 2 3 4 5
49. Frustriranost zato što može obaviti manje stvari nego što je prije mogao.	1 2 3 4 5	1 2 3 4 5
50. Doživljaj gubitka kontrole nad vlastitim tijelom.	1 2 3 4 5	1 2 3 4 5
51.Doživljaj gubitka kontrole nad vlastitim životom.	1 2 3 4 5	1 2 3 4 5
52. Poteškoće pri traženju pomoći.	1 2 3 4 5	1 2 3 4 5
53. Poteškoće pri donošenju vlastitih odluka.	1 2 3 4 5	1 2 3 4 5

Psychological, Social, and Spiritual Problems of Palliative Patients' Questionnaire

Effectiveness in Coping with the Psychological, Social, and Spiritual Challenges of Palliative Care Patients

The aim of this study is to explore nurses' perceptions of the needs and satisfaction of palliative care patients. The research is completely anonymous, and your participation is voluntary; you may withdraw at any time. By completing the questionnaire, you agree to participate in the research.

The terms used in the questionnaires have gender meanings, regardless of whether they are used in the masculine or feminine form, they are neutral and encompass both genders equally.

Instructions for completing the questionnaires:
These questionnaires are intended for nurses who work with palliative patients. Please ensure that your responses pertain only to working with patients who suffer from:
• acute, serious, and life-threatening diseases where treatment is very prolonged, demanding, and accompanied by significant difficulties, but cure is still possible;
• progressive chronic diseases and conditions (malignant diseases, chronic diseases), dementia, and other illnesses that cause complete dependence in satisfying basic human needs;
• conditions caused by severe injuries and traumas;
• diseases whose stage is in the final phase (dementia, terminal stage of cancer, other diseases or severe disability) where recovery is not possible, and care is essential until the end of life.

Your occupation _________________________

Institution/department where you are employed ______________________________

Experience working with palliative patients (number of years)_______________

The questionnaire consists of two parts:

In the first section, questions relate to your perception and experience of working with palliative patients. Please answer the questions with YES or NO.

1. Working with palliative patients is part of my regular job duties. YES NO
2. During my formal education, I attended lectures and took exams in the field of palliative care. YES NO
3. After completing my formal education, I participated in lifelong learning programs in the field of palliative care. YES NO
4. I consider myself adequately educated in the field of palliative care. YES NO
5. Working with palliative patients is very emotionally draining for me, and sometimes I need to help myself to better respond to the problems/needs of palliative patients. YES NO
6. Professionals working in palliative care need various forms of support due to the emotional demands of the job, including psychological assistance. YES NO
7. I believe that more subjects related to palliative medicine should be introduced into the programs of secondary health schools and faculties. YES NO
8. I believe that it is necessary to organize more events where knowledge in the field of palliative medicine will be presented. YES NO
9. Palliative care should be provided by an interdisciplinary team of experts (e.g., doctors, nurses, psychologists, clergy, social workers, etc.). YES NO

10. In our healthcare and social care system, there are palliative care teams that are sufficient to care for palliative patients. YES NO
11. The care of palliative patients' needs is mostly left to families. YES NO

Second Part: Please indicate in the second column the extent to which most individuals receiving palliative care experience difficulties or have needs in various areas of their lives. Circle the number (from 1 to 5) that reflects **your opinion** on the severity of each listed difficulty.

In the second column, circle the number (from 1 to 5) that reflects **your opinion** on the extent to which these needs are met.

**Difficulties/needs**	To what extent do most palliative patients experience difficulties or have needs in different areas of their lives?<br>1 - Not a problem at all<br>2 - Difficulty/need is present to a lesser extent<br>3 - Difficulty/need is moderately expressed<br>4 - Difficulty/need is very pronounced<br>5 - It is the greatest difficulty/unmet need for most palliative care users	To what extent are the needs of most palliative patients met?<br>1 - Not satisfied at all<br>2 - Satisfied to a lesser extent<br>3 - Need is satisfied to a moderate degree<br>4 - Satisfied to a greater extent<br>5 - Completely satisfied
1. Difficulty in planning the day.	1 2 3 4 5	1 2 3 4 5
2. Difficulty in relaxing.	1 2 3 4 5	1 2 3 4 5
3. Difficulty in finding employment or continuing education (does not apply to patients with dementia or in the terminal stage).	1 2 3 4 5	1 2 3 4 5
4. Difficulty in caring for children or childcare (does not apply to patients with dementia or in the terminal stage).	1 2 3 4 5	1 2 3 4 5
5. Problems in the relationship with the partner.	1 2 3 4 5	1 2 3 4 5
6. Difficulty in discussing illness with the partner.	1 2 3 4 5	1 2 3 4 5
7. Problems in the relationship with children (or with only one child).	1 2 3 4 5	1 2 3 4 5
8. Difficulty in relationships with family, friends, neighbors, or colleagues.	1 2 3 4 5	1 2 3 4 5
9. Difficulty in talking about illness because of not wanting to burden others.	1 2 3 4 5	1 2 3 4 5
10. Belief that others are not ready to discuss the illness.	1 2 3 4 5	1 2 3 4 5
11. Differences of opinion regarding the type of treatment to be applied.	1 2 3 4 5	1 2 3 4 5
12. Experiences insufficient support from others.	1 2 3 4 5	1 2 3 4 5
13. Difficulty in finding a trustworthy person to talk to about the illness.	1 2 3 4 5	1 2 3 4 5

14. Receives insufficient practical help from the partner or family.	1 2 3 4 5	1 2 3 4 5
15. Others behave overly concerned.	1 2 3 4 5	1 2 3 4 5
16. Other significant people dramatize the situation.	1 2 3 4 5	1 2 3 4 5
17. Others deny the seriousness of the situation.	1 2 3 4 5	1 2 3 4 5
18. Loneliness.	1 2 3 4 5	1 2 3 4 5
19. Others have abandoned him/her.	1 2 3 4 5	1 2 3 4 5
20. Depressive mood.	1 2 3 4 5	1 2 3 4 5
21. No longer feels pleasure in anything.	1 2 3 4 5	1 2 3 4 5
22. Fear of physical suffering.	1 2 3 4 5	1 2 3 4 5
23. Fear of medical treatment.	1 2 3 4 5	1 2 3 4 5
24. Fear of worsening of the illness.	1 2 3 4 5	1 2 3 4 5
25. Fear of loneliness.	1 2 3 4 5	1 2 3 4 5
26. Fear of death.	1 2 3 4 5	1 2 3 4 5
27. Difficulty in coping with the unpredictability of the future.	1 2 3 4 5	1 2 3 4 5
28. Difficulty in expressing emotions.	1 2 3 4 5	1 2 3 4 5
29. Feelings of guilt.	1 2 3 4 5	1 2 3 4 5
30. Feelings of shame.	1 2 3 4 5	1 2 3 4 5
31. Loss of control over emotions.	1 2 3 4 5	1 2 3 4 5
32. Difficulty in accepting changes in physical appearance.	1 2 3 4 5	1 2 3 4 5
33. Difficulty in seeing the positive aspects of the situation.	1 2 3 4 5	1 2 3 4 5
34. Feeling overwhelmed by all the decisions that need to be made.	1 2 3 4 5	1 2 3 4 5
35. Difficulty in engaging in meaningful purposes.	1 2 3 4 5	1 2 3 4 5
36. Difficulty in being available to others.	1 2 3 4 5	1 2 3 4 5
37. Difficulty in maintaining trust in God or religion.	1 2 3 4 5	1 2 3 4 5
38. Difficulty in finding meaning in death.	1 2 3 4 5	1 2 3 4 5
39. Difficulty in accepting the illness.	1 2 3 4 5	1 2 3 4 5
40. Difficulty in accepting lost time or missed opportunities in life.	1 2 3 4 5	1 2 3 4 5
41. Difficulty in accepting wrong life choices that cannot be corrected.	1 2 3 4 5	1 2 3 4 5
42. Difficulty due to the inability to reconcile with certain people who have passed through their lives.	1 2 3 4 5	1 2 3 4 5

43. Difficulty in seeking spiritual assistance due to fear of death.	1 2 3 4 5	1 2 3 4 5
44. Difficulty in receiving the sacrament of the anointing of the sick due to associating it with last rites or imminent death.	1 2 3 4 5	1 2 3 4 5
45. Difficulty in performing daily activities.	1 2 3 4 5	1 2 3 4 5
46. Difficulty in resuming social activities.	1 2 3 4 5	1 2 3 4 5
47. Difficulty in delegating tasks to others due to inability to continue performing them.	1 2 3 4 5	1 2 3 4 5
48. Difficulty due to dependence on others.	1 2 3 4 5	1 2 3 4 5
49. Frustration because they can do fewer things than they could before.	1 2 3 4 5	1 2 3 4 5
50. Feeling of losing control over their own body.	1 2 3 4 5	1 2 3 4 5
51. Feeling of losing control over their own life.	1 2 3 4 5	1 2 3 4 5
52. Difficulty in seeking help.	1 2 3 4 5	1 2 3 4 5
53. Difficulty in making their own decisions.	1 2 3 4 5	1 2 3 4 5
